# A standardized protocol for the multiplex PCR technique Septifast^® ^Roche for neonatal samples with suspected sepsis

**DOI:** 10.1186/cc11766

**Published:** 2012-11-14

**Authors:** F Ortiz Ibarra, J Reyna, P Treviño, L Fernandez, G Lara, E Valenzuela, Y Morales, A Limon, A Ceballos

**Affiliations:** 1 Laboratorios Diagnomol, Mexico; 2 INSP, Mexico; 3 HMI SSNL, Monterrey, Mexico; 4 INPerIER, Mexico; 5 HGO4 IMSS, Mexico; 6 HAE Pemex Sur, Mexico; 7 H Dalinde, Mexico

## Introduction

High morbidity and mortality rates are associated with sepsis in newborns, as well as low recovery rates, extended recovery times, and delayed culture times of blood culture. To surmount these parameters, new molecular techniques are required by the clinical laboratory. The LightCycler Septifast^® ^technique, which identifies up to 25 microorganisms known to cause over 90% of admissions to ICUs due to infection, has proven its usefulness in adult patients in several countries. The objective was to standardize the Septifast^® ^Roche technique for diagnostic use in newborns with suspected sepsis.

## Methods

Eighty-six newborn samples with suspected sepsis (according to the NOSEP-1 scale) were included. We analyzed two blood samples per patient, the first one collected in an EDTA-anticoagulated tube (0.5 to 1.0 ml), and a second sample of 1 cm^3 ^in a hemoculture tube for automated hemoculture procedure using BacT/ALERT^®^3D (Biomerieux). Briefly, the whole sample was lysed with MagNA Lyser (Roche), and nucleic acid purification was performed with Septifast prep M^grade ^(Roche). DNA from Gram-negative, Gram-positive, and fungi was detected using multiplex LightCycler 2.0 real-time methodology with LightCycler SeptiFast M^grade ^kit (Roche). Finally, results analysis was performed with Roche Septifast identification software (SIS).

## Results

Of the 86 samples analyzed, 31 (36.04%) were positive in at least one of the identification procedures. Thirteen samples (15.11%) were rendered positive by hemoculture, and 27 (31.39%) by Septifast^® ^protocol. Fifty-seven samples (66.20%) were negative by both analytic procedures, and a total of 31 pathogens (15 Gram-negative, 11 Gram-positive and five fungi species) were identified by Septifast^® ^(four of them in combination) versus nine species recovered from hemocultures. The response time ranged from 6 to 24 hours by Septifast^® ^procedure, compared with 4 to 7 days by hemoculture detection.

## Conclusion

The use of the multiplex real-time PCR Septifast^® ^technique allows one to detect a wider number of pathogenic species and a faster response time than hemoculture, with a small quantity of blood sample (1 ml).

